# Fascin overexpression promotes neoplastic progression in oral squamous cell carcinoma

**DOI:** 10.1186/1471-2407-12-32

**Published:** 2012-01-20

**Authors:** Hunain Alam, Amruta V Bhate, Prakash Gangadaran, Sharda S Sawant, Shimul Salot, Lalit Sehgal, Prerana P Dange, Devendra A Chaukar, Anil K D'cruz, Sadhna Kannanl, Rajiv Gude, Shubhada Kane, Sorab N Dalal, Milind M Vaidya

**Affiliations:** 1 Advanced Centre for Treatment Research and Education in Cancer Tata Memorial Centre (ACTREC), Kharghar, Navi Mumbai, India-410210; 2 Oral Surgery, Head and Neck Unit, Tata Memorial Hospital, Parel, Mumbai, India-400012; 3 Dept. of Pathology, Tata Memorial Hospital, Parel, Mumbai, India-400012

**Keywords:** Fascin, Cell migration, Invasion, Metastasis, OSCC

## Abstract

**Background:**

Fascin is a globular actin cross-linking protein, which plays a major role in forming parallel actin bundles in cell protrusions and is found to be associated with tumor cell invasion and metastasis in various type of cancers including oral squamous cell carcinoma (OSCC). Previously, we have demonstrated that fascin regulates actin polymerization and thereby promotes cell motility in K8-depleted OSCC cells. In the present study we have investigated the role of fascin in tumor progression of OSCC.

**Methods:**

To understand the role of fascin in OSCC development and/or progression, fascin was overexpressed along with vector control in OSCC derived cells AW13516. The phenotype was studied using wound healing, Boyden chamber, cell adhesion, Hanging drop, soft agar and tumorigenicity assays. Further, fascin expression was examined in human OSCC samples (N = 131) using immunohistochemistry and level of its expression was correlated with clinico-pathological parameters of the patients.

**Results:**

Fascin overexpression in OSCC derived cells led to significant increase in cell migration, cell invasion and MMP-2 activity. In addition these cells demonstrated increased levels of phosphorylated AKT, ERK1/2 and JNK1/2. Our in vitro results were consistent with correlative studies of fascin expression with the clinico-pathological parameters of the OSCC patients. Fascin expression in OSCC showed statistically significant correlation with increased tumor stage (*P *= 0.041), increased lymph node metastasis (*P *= 0.001), less differentiation (*P *= 0.005), increased recurrence (*P *= 0.038) and shorter survival (*P *= 0.004) of the patients.

**Conclusion:**

In conclusion, our results indicate that fascin promotes tumor progression and activates AKT and MAPK pathways in OSCC-derived cells. Further, our correlative studies of fascin expression in OSCC with clinico-pathological parameters of the patients indicate that fascin may prove to be useful in prognostication and treatment of OSCC.

## Background

Oral squamous cell carcinoma (OSCC) is the sixth most common malignancy in the world and ranks as the first in males in the Indian subcontinent. It is a major cause of cancer morbidity and mortality [1]. Unfortunately, despite the advancements in surgery, chemotherapy, radiation and other combinational therapies, only 60% of affected individuals survive for 5 years [2,3]. Local recurrence and regional lymph node metastasis are two major hurdles in the management of the advanced stage OSCC [4-6]. Thus a comprehensive investigation of the factors and molecular events which contribute to local recurrence and invasion of OSCC are necessary for the development of novel strategies for prognostication and treatment.

Metastatic and invasive tumor cells often exhibit changes in cell morphology, disruption of cell-cell contacts, degradation of ECM and increase in cell migration, which result from rearrangements of the cytoskeletal microfilaments. Reorganization of the actin cytoskeleton is regulated by the action of actin cross-linking proteins. Fascin is a highly conserved 55-kDa actin bundling protein that plays an important role in the organization of several types of actin-based structures such as filopodia, lamellipodial ribs, dendrites, spikes and microvilli [7]. It was first detected in the extracts of unfertilized sea urchin eggs and localized within microvilli and filopodia of fertilized sea urchin eggs [8]. Fascin is predominantly expressed in cells which form membrane protrusions and require motility, such as neurons, glial cells and dendritic cells [9-11] and also in migrating cells such as endothelial cells and macrophages [11]. Fascin expression is either low or absent in adult epithelia and is often upregulated in several types of epithelial cancers [12] including breast [13,14], ovary [15], skin [16], pancreas, liver cancer etc. [17-19]. High expression of fascin has also been reported in OSCC. Fascin is also found to be involved in formation of invadopodia and appears to aid tumor cell invasion [20]. A number of prior studies have shown that fascin upregulation is associated with a more aggressive and metastatic phenotype in epithelial cancers [12,21-27]. Although, several correlative studies have demonstrated tumor promoting function of fascin, its role in tumor development and/or progression of OSCC has not been comprehensively investigated yet.

We have previously reported that fascin regulates actin polymerization and cell motility in K8-knockdown OSCC cells. Decrease in fascin levels was also associated with reduced invasive ability and tumorigenicity in K8-depleted cells [28]. In the present communication, we have demonstrated the role of fascin in cell migration, invasion and tumorigenicity in fascin overexpressed-OSCC-derived cell line. Further, we show a higher expression of fascin in tissue samples of OSCC using IHC analysis, which correlated with clinico-pathological parameters of the patients such as tumor stage, lymph node metastasis and survival.

## Methods

### Cell lines, plasmids, transfection, western blotting and immunofluorescence staining

The cell line AW13516 derived from the SCC of tongue was cultured as described previously [28]. The fascin overexpressed clones were selected in 1000 μg/ml G418 sulphate, from AW13516 cells by transfecting 2 μg of GFP tagged fascin (a kind gift from Dr. J Adams, USA), or the empty vector, using liposome based FUGENE HD transfection reagent (Roche; according to manufacturer's protocol). Stable clones were screened for the overexpression of fascin by laser confocal microscopy and western blot analysis as described previously [29].

### Antibodies and reagents

The following antibodies were used -Keratin8 and β-actin (Sigma), β4-integrin, Akt and p38 (Santa Cruz), phospho-JNK1/2, JNK1/2, phospho-FAK, phospho-p38 and FAK (Cell Signalling), fascin, β-catenin, phospho-Akt, phospho-ERK1/2, and ERK1/2 (Abcam), E-cadherin and PCNA (BD Biosciences), secondary antibodies- HRP conjugated anti-mouse and anti-rabbit (Amarsham), Alexa 488-conjugated anti-mouse IgG and Alexa 568-conjugated anti-rabbit IgG (Molecular Probes). TRITC conjugated phalloidin (Sigma) was used to analyze filamentous actin according to the manufacturer's instructions.

### Cell proliferation assay (MTT assay)

Proliferation rate was determined by taking OD every 24 h up to a period of 5 days using MTT assay as described previously [30].

### Hanging drop assay

Hanging drop assay was performed to estimate the cell-cell adhesive properties of the fascin-overexpressed cells compared to the vector control clones as described previously [31]. Briefly, 2 × 10^4 ^cells were suspended in 35 μl drops of complete medium from the lid of 24-well plate for 16 h. Images of cell aggregates of five random fields from three different suspensions were captured with an upright AxioImager. Z1 microscope (Carl Zeiss, Germany) for each sample. The area of cell aggregates was determined using the Axiovision rel 4.5 software (Zeiss).

### Cell adhesion assay

96 well flat bottom plates coated with 50 μl/well of ECM substrates (Matrigel: 10 μg/ml, Fibronectin: 2.5 μg/ml, Collagen type IV: 10 μg/ml, Laminin: 5 μg/ml in PBS) and cell-ECM adhesion assay was carried out as described previously [32].

### Zymography

To determine the Matrix Metalloproteinase (MMP) activity in conditioned culture medium, gelatin zymography was performed as described previously [33]. The position of the MMPs was visualized by staining the gels in Coomassie Brilliant Blue G-250 solution [33].

### Tumorigenicity, invasion and wound-healing assay

Soft agar assay and tumor formation in SCID mice was performed as described previously [28]. Five animals were used for each group. The ellipsoid volume formula 1/2 × L × W × H was used to calculate the tumor volume. The invasiveness was determined by Boyden chamber invasion assay as described previously [32]. Briefly, 4 × 10^4 ^cells in 200 μl of cell suspension were charged on to the insert and the plates were incubated for 40 h at 37°C. Post incubation the total number of cells was counted in 4-5 different fields. Data obtained from three separate chambers were shown as mean values. Wound-healing assay was performed as described previously [28]. Briefly, the cells were grown in 35 mm plates upto 80% confluency and were replaced with serum free medium to inhibit cell proliferation. After 16 h of serum starvation, wounds were scratched with the help of sterile 2 μl pipette tip. The wound healings were observed by time lapse microscopy, and images were taken every 10' for 12 h. Migration was measured using Axiovision software version 4.5 (Zeiss).

### Surgical specimens and clinico-pathological records of the patients

A total of 131 paraffin embeded blocks of oral SCC [71 SCC of tongue (TSCC) and 60 SCC of buccal mucosa (BMSCC)] tissues were collected from the Tata Memorial Hospital (TMH), Parel, Mumbai, India. Six OSCC tissues (primary tumors as well as lymph nodes) of patients showing lymph node metastasis were collected from operation theatre of TMH at the time of surgery. Five punch biopsy samples of patients suffering from inflammatory fibrous hyperplasia of oral cavity (2 BM and 3 Gingival) were obtained from Nair Dental College, Mumbai, India as controls. The patients had no history of malignancy. This study was approved by the Human Ethics Committees of the respective Institutional Review Boards. Informed consent was obtained from the patients as well as healthy individuals. Clinico-pathological information was collected from the clinical and pathology records of TMH (Additional file 1: Table S1).

### Histology, immunohistochemistry (IHC) and immunofluorescence (IF)

Tissue samples were fixed in 10% formalin buffer and five micron sections were cut from paraffin embedded blocks. Sections were stained with hematoxylin/eosin for histological diagnosis. Immunohistochemical staining was performed using Elite ABC Kit (Vector laboratories; USA) as described earlier [34]. The primary antibodies, fascin (mouse anti-human; Abcam), K8 (mouse anti-human; Sigma) and β4-integrin (rabbit anti-human; Santacruz Biotechnology) were used at dilutions of 1:100, 1:200 and 1:100 respectively. Specimens for fascin, K8 and β4-integrin immunostaining were divided into three categories: High (homogenous positive tumor in more than 50% of cells or/and high membranous and cytoplasmic staining in more than 50% of cells; ++/+++); low (20-50% positive tumor cells; +); negative (less than 20% positive cells or no evidence of staining; -). Results of IHC were independently assessed by two observers. For immunofluorescence staining Alexa 488-conjugated anti-mouse IgG (1:200) and Alexa 568-conjugated anti-rabbit IgG (1:200) were used as secondary antibodies. The tissue sections were mounted using VECTASHEILD mounting medium (Vector Laboratories). Images were captured using laser confocal microscopy.

### Statistical analysis

Statistical analysis of the clinical samples was carried out using SPSS software. To assess correlations between clinico-pathological parameters and proteins expression, the Chi Square test was used. Univariant analysis was performed using the Kaplan-Meier method and statistical significance between survival curves was assessed by the log rank tests. The data were analyzed with the Statistical Package, SPSS 16.0 for Windows (SPSS Inc., Chicago, IL, USA) [34]. Two groups of data were statistically analyzed by *t test *using Graphpad Prism5 Software. A p value less than 0.05 was considered statistically significant.

## Results

### Fascin overexpression leads to alterations in cell morphology and actin organization

**Figure 1:**
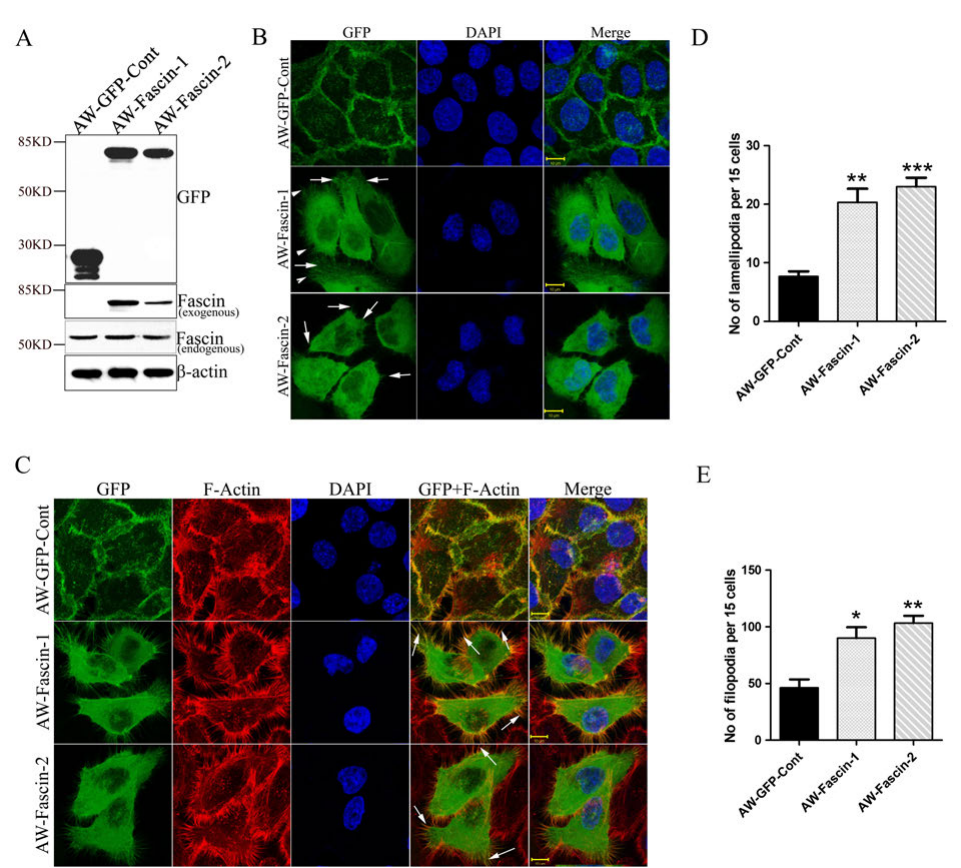
**Overexpression of fascin leads to reorganization of actin cytoskeleton**. (**A**) Western blot and confocal analysis of stable overexpression of Fascin-GFP (AW-Fascin-1 and AW-Fascin-2) and empty vector control pEGFP (AW-GFP-Cont) clones derived from AW13516 cells with antibodies to GFP and fascin. β-actin was used as loading control. Scale bars: 10 μm. (**B**) Representative confocal images of stably expressed GFP tagged fascin and GFP alone in AW-Fascin-1, AW-Fascin-2 and AW-GFP-Cont clones respectively. Cells were counter stained with DAPI. Scale bars: 10 μm. Arrow heads and arrows indicate filopodia and lamellipodia respectively. (**C**) Representative confocal images of F-actin stained with phalloidin-TRITC and the co-localization of F-actin with Fascin-GFP in stable AW-Fascin-1, AW-Fascin-2 and AW-GFP-Cont clones. Cells were counter stained with DAPI. Scale bars: 10 μm. Arrows indicate colocalization of Fascin-GFP with F-actin at filopodia like structures. (**D**) Histogram showing number of filopodia formed by fascin overexpressed (AW-Fascin-1 and AW-Fascin-2) and vector control (AW-GFP-Cont) cells. Mean and standard deviation of 3 independent experiments is plotted (*p *< 0.0001). (**E**) Histogram showing number of lamellipodia formed by fascin overexpressed (AW-Fascin-1 and AW-Fascin-2) and vector control (AW-GFP-Cont) cells. Mean and standard deviation of 3 independent experiments is plotted (*p *< 0.0001)

In order to understand the role of fascin in progression of OSCC, GFP tagged fascin or pEGFP empty vector was stably-overexpressed in OSCC derived cell line AW13516. Stable over expression of exogenous fascin and GFP was checked by western blotting and confocal microscopy (Figure 1A, B). Exogenous fascin was found to be localized on the cell membrane as well as in the cytoplasm. Membranous fascin appeared mostly in the form of microspikes and intermittently was present on the edges of the cell membrane (Figure 1B). Morphological alterations like increase in membrane protrusions and disorganization of cell-cell contacts were observed by phase contrast microscopy in fascin-overexpressed cells. (Additional file 2: Figure S1A). Further, to determine the effect of fascin overexpression on organization of actin cytoskeleton, these cells were stained with TRITC-labelled phalloidin and were analyzed by confocal microscopy. Fascin overexpressing clones AW-Fascin-1 and AW-Fascin-2 demonstrated dramatic increase in membrane protrusions like filopodia and lamellipodia compared to the vector control clone AW-GFP-Cont (Figure 1C, D). In addition, exogenous fascin was found to be co-localized with F-actin at mostly at membrane protrusions and filopodia (Figure 1A). These results together indicate that fascin overexpression leads to increase in F-actin based structures like filopodia and lamellipodia. The changes observed in actin reorganization also correlated well with morphological alterations exhibited by fascin overexpressed cells.

### Fascin overexpression leads to increase in cell migration, invasion and matrix metalloproteinase activity

**Figure 2:**
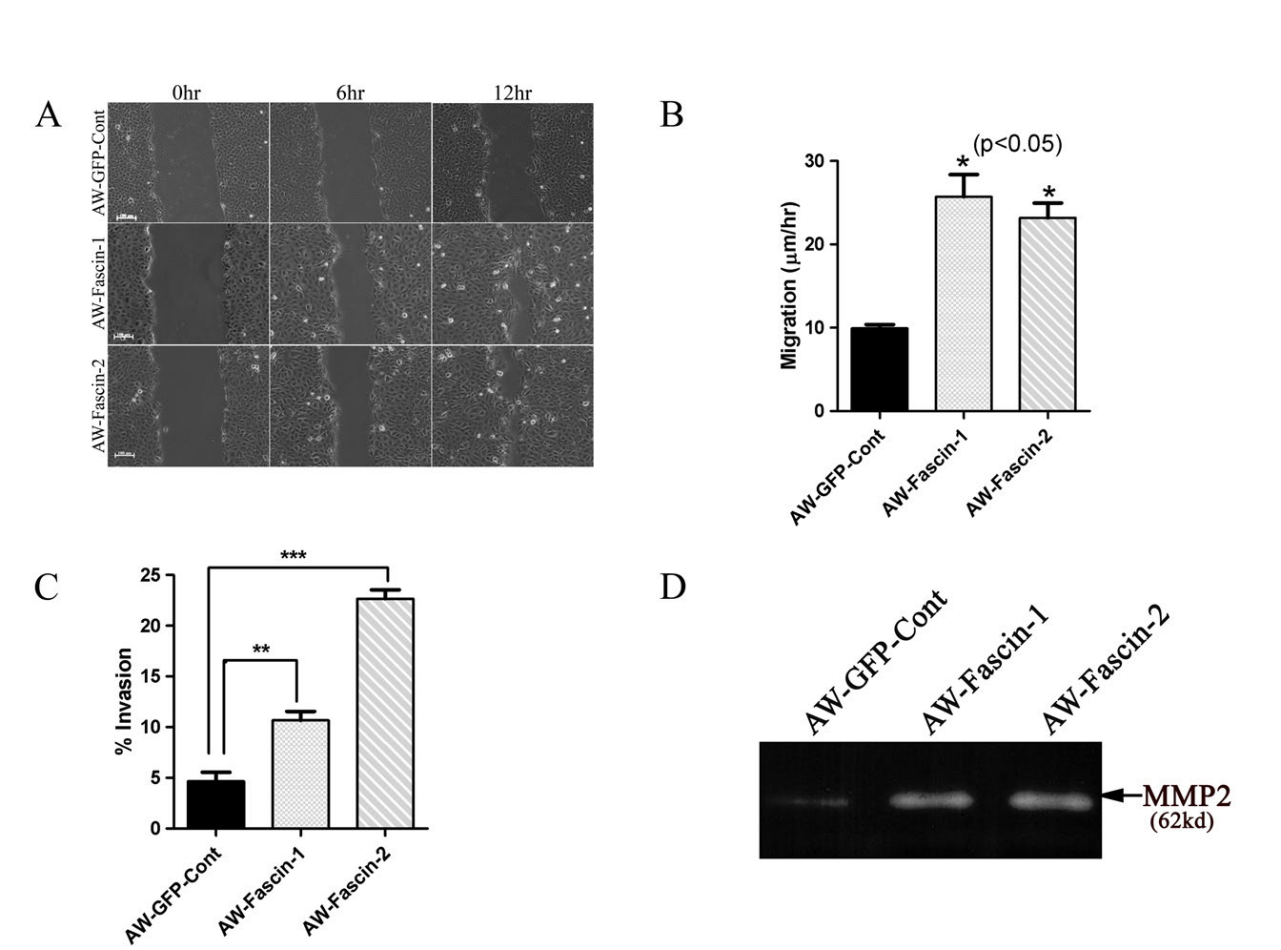
**Overexpression of fascin leads to increase in cell migration, invasion and MMPs activity**. (**A **and **B**) Phase contrast images (magnification 10×) of wound closure at 0 h, 6 h and 12 h of the vector control (AW-GFP-Cont) and fascin overexpressed clones (AW-Fascin-1 and AW-Fascin-2) are shown in the figure. Scale bar: 100 μm. The data shown is the average migration rate from three independent experiments with the mean and standard deviation (*p *< 0.05). (**C**) Boyden chamber invasion assay of vector control (AW-GFP-Cont) and fascin overexpressed clones (AW-Fascin-1 and AW-Fascin-2). The data shown is the average from three independent experiments with the mean and standard deviation (*p *< 0.01). (**D**) Zymogram showing MMP-2 activity in vector control (AWGFP-Cont) and fascin overexpressed (AW-Fascin-1 and AW-Fascin-2) cells.

To determine if the alterations in actin organization and morphology upon fascin overexpression led to changes in cell migration and invasion, scratch wound healing assay and Boyden chamber invasion assay were performed respectively (Figure 2A). Fascin overexpressed clones (AW-Fascin-1and AW-Fascin-2) showed more than two fold increase in cell migration rate compared to vector control (AW-GFP-Cont.) clone (*P *< 0.05; Figure 2B). These cells also demonstrated significant increase in invasive ability as compared to vector control cells (*P *= 0.01; Figure. 2C). In addition, results of our gelatin zymography experiments showed that fascin overexpressed clones secreted more MMP2 compared to control cells (Figure 2D). These data together suggest that, increase in filopodia formation and actin reorganization upon fascin overexpression is associated with an increase in migratory and invasive ability of these cells.

### Fascin overexpression resulted in increased cell-ECM adhesion and loss of cell-cell contacts

**Figure 3:**
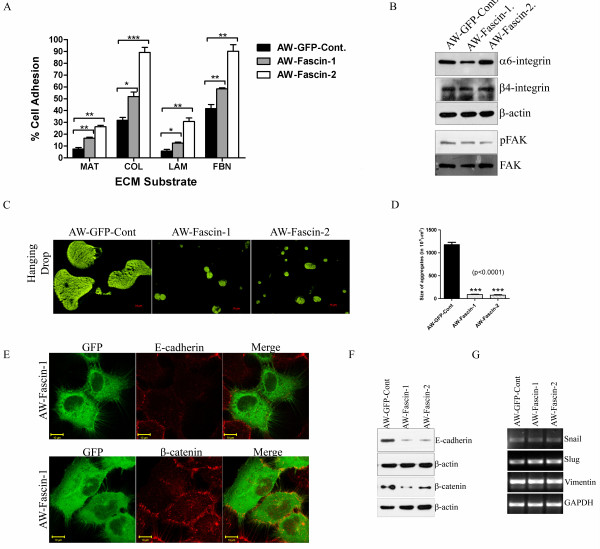
**Fascin overexpressed cells demonstrated increase in cell-ECM adhesion and loss of cell-cell contacts**. (**A**) Cell adhesion of fascin-overexpressed (AW-Fascin-1 and AW-Fascin-2) and vector control (AW-GFP-Cont) cells to different ECM substrates was measured as described. The data shown is the average from three experiments with the mean and standard deviation. (**B**) Western blot analysis of stable fascin-overexpressed (AW-Fascin-1 and AW-Fascin-2) and vector control (AW-GFP-Cont) clones with antibodies to α6 integrin, β4 integrin and phosphorylated FAK. β-actin and total FAK were used as internal loading controls respectively. (**C**) Representative images of aggregates formed by of fascin overexpressed (AW-Fascin-1 and AW-Fascin-2) and vector control (AW-GFP-Cont) cells in hanging drop assay. (**D**) Histogram showing size of aggregates formed by overexpressed (AW-Fascin-1 and AW-Fascin-2) and vector control (AW-GFP-Cont) cells. Mean and standard deviation of 3 independent experiments is plotted (*p *< 0.0001). (**E**) Confocal analysis of E-cadherin and β-catenin localization in fascin overexpressed cells. Scale bars; 10 μm. (**F**) Western blot analysis of fascin overexpressed (AW-Fascin-1 and AW-Fascin-2) and vector control clones (AW-GFP-Cont) with antibodies to E-cadherin and β-catenin. β-actin was used as a loading control. (**G**) RT-PCR analysis of snail, slug and vimentin overexpressed (AW-Fascin-1 and AW-Fascin-2) and vector control (AW-GFP-Cont) cells. GAPDH was used as internal control.

Since filopodia have an important role to play in cell-ECM adhesion, we checked whether fascin overexpression affects OSCC cell adhesion. Fascin-overexpressed clones demonstrated significant increase in adhesion to different ECM substrates such as matrigel, collagen, laminin and fibronectin in comparison with vector control cells (Figure 3A). We did not observe any considerable change in α6 and β4 integrin levels upon fascin overexpression. There was no considerable change in the levels of phosphorylated FAK in fascin overexpressed cells (Figure 3B).

Our observations using phase contrast microscopy suggest that fascin overexpression in the cells may result in loss of cell-cell contacts. In order to confirm this observation, hanging drop assay was performed. The fascin overexpressed clones formed smaller aggregates compared to the vector control clone which indicates that fascin overexpression leads to loss of cell-cell contacts (*P *< 0.0001) (Figure 3C, D; Additional file 3: Table S2). Further, we examined whether the level of expression of β-catenin and E-cadherin was altered in fascin-overexpressed cells using western blot analysis. There was significant reduction in E-cadherin and β-catenin levels in these cells. We also observed colocalization of β-catenin with fascin in fascin overexpressing cells (Figure 3E, F) and reduction in their levels at the cell surface (Additional file 2: Figure S1C) as analyzed by confocal microscopy.

Since fascin overexpressed cells showed significant increase in cell migration, invasion and substantial reduction in E-cadherin levels which are known to be associated with EMT, we also determined the levels of EMT markers such as snail, slug and vimentin using RT-PCR analysis. Fascin overexpressed cells did not show any increase in these markers (Figure 3G). Further, we also analyzed the protein levels of vimentin in these clones by western blot analysis. There was no significant difference observed at the protein levels of vimentin between these clones (Additional file 2: Figure S1B). These results indicate that overexpression of fascin led to increase in cell-ECM adhesion and loss of cell-cell contacts.

### Fascin overexpression leads to increase in tumorigenicity both in vitro and in vivo

**Figure 4:**
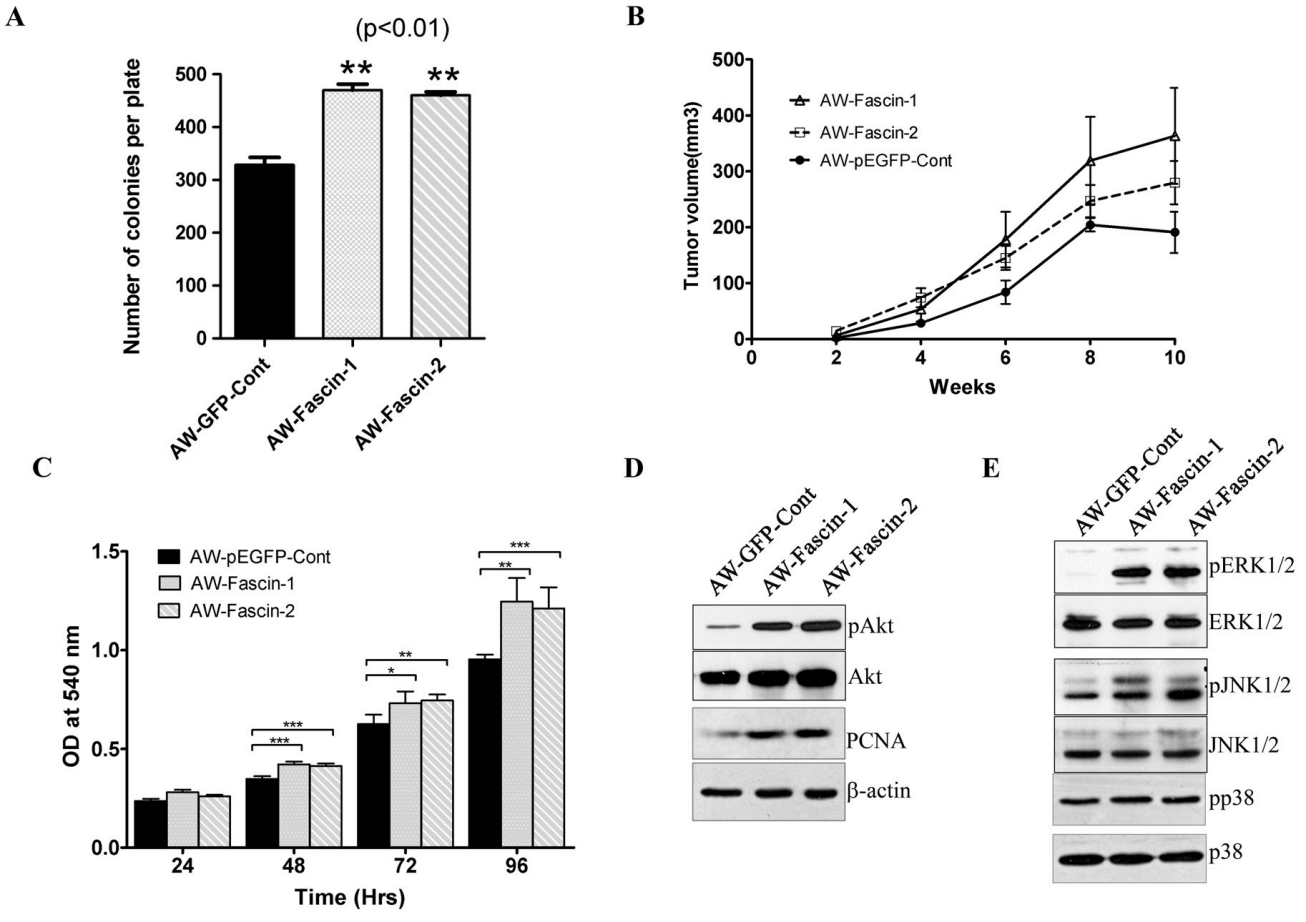
**Fascin overexpression resulted in increase in cell proliferation and tumorigenicity**. (**A**) Histogram showing the total number of colonies formed per plate by the indicated clones after 14 days. Mean and standard deviation of three independent experiments is plotted (*p *< 0.01). (**B**) Tumor growth of fascin-overexpressed clones (AW-Fascin-1, AW-Fascin-2) or the vector control (AW-GFP-Cont) was plotted against time interval. (**C**) Cell proliferation curves of fascin-overexpressed (AW-Fascin-1 and AW-Fascin-2) and vector control (AW-GFP-Cont) cells using MTT assay. The cell number was determined at the times indicated. Each point represents the mean and the standard errors of three independent experiments done in triplicate. (**D** and **E**) Western blot analysis of stable fascin-overexpressed (AW-Fascin-1 and AW-Fascin-2) and vector control (AW-GFP-Cont) clones with antibodies to PCNA and phosphorylated forms of Akt, ERK1/2, JNK1/2 and p38. Total Akt, ERK1/2, JNK1/2, p38 and β-actin were used as internal loading controls respectively.

To determine the effect of fascin expression on tumorigenicity in vitro and in vivo, anchorage-independent growth and tumor formation in SCID mice was studied. The fascin-overexpressed clones (AW-Fascin-1 and AW-Fascin-2) demonstrated significant increase in number (Figure 4A) and size of colonies formed in soft agar compared to AW-GFP-Cont after 14 days (*P *< 0.01) (Additional file 2: Figure S1D). We compared the growth of subcutaneous xenografts of fascin-overexpressed clones and vector control clone in NOD-SCID mice for 10 weeks. Both AW-Fascin-1 and AW-Fascin-2 clones showed considerable increase in tumor volume compared to AW-GFP-Cont clone after 10 weeks (*P *= 0.06) (Figure 4B).

### Fascin overexpression leads to increase cell proliferation and upregulation of MAP kinase and AKT pathways

To check the effect of fascin expression on cell proliferation, cell proliferation rate of fascin overexpressed and vector control cells was analyzed by MTT asaay. Fascin-overexpressed cells showed significant increase in cell proliferation as compared to vector control cells (Figure 4C). Furthermore, we observed substantial increase in cell proliferation marker PCNA using western blot analysis (Figure 4D). To determine whether the overexpression of fascin modulates cell proliferation and/or oncogenic signalling pathways, some central regulatory molecules of MAP kinase and PI3K signalling pathways were examined in these cells using western blot analysis. A significant increase was observed in the levels of phosphorylation of both ERK1/2 and JNK1/2 in the fascin overexpressed cells (MAP kinase signalling pathway) using western blot analysis (Figure 4E). Fascin overexpressed cells also demonstrated enhanced phosphorylation of Akt compared to vector control cells (PI3K signalling pathway) (Figure 4D). We also analyzed the phosphorylation levels of p38 in these cells and no change was observed in the levels of phosphorylated p38. The increased phosphorylation of ERK1/2, JNK1/2 and Akt correlated with the increased cell proliferation, motility and tumorigenicity upon fascin overexpression.

### Expression of fascin in human OSCC samples

Fascin expression was analyzed in 131 OSCC tissue samples using semi-quantitative IHC analysis. Out of 131 samples studied, 43 (32.28%) samples showed intense fascin staining, 55 (41.98%) showed weak staining of fascin and it was not detected in 33 (25.19%) samples (Table 1). Fascin staining was observed both in cytoplasm and cell membrane (Additional file 4: Figure S2A). Fascin expression was also analyzed in 5 samples obtained from oral mucosa showing only inflammatory changes. Weak or negative staining of fascin was seen in these samples (Additional file 4: Figure S2B). We have also confirmed the expression of fascin in normal as well as tumor tissues using immunofluorescence staining followed by laser confocal analysis (Additional file 5: Figure S3).

**Table 1:** Correlation of fascin expression with clinico-pathological parameters of the OSCC patients.

Clinico-pathological parameter		FASCIN				
		
		n(131),%	EXPERSSION			*P*-value
				
			Negative	Low	High	
Age (Years)	< 50	75 (57.25)	15	30	30	0.091*
		
	≥50	56 (42.75)	18	25	13	

Sex	Male	102 (77.87)	25	41	36	0.524*
		
	Female	29 (22.14)	8	14	7	

Location	Tongue	61 (46.56)	17	27	26	0.517*
		
	BM	70 (53.44)	16	28	17	

Thickness	< 2 cm	92 (70.23)	25	35	32	0.370*
		
	≥2 cm	39 (29.77)	8	20	11	

Stages	I/II	28 (21.38)	9	15	4	0.041^#^
		
	III/IV	103 (78.62)	24	40	39	

Tumor Size	T1/T2	48 (36.65)	13	23	12	0.254^#^
		
	T3/T4	83 (63.35)	20	31	32	

Node Status	NO	47 (35.88)	17	22	8	0.001^#^
		
	N1	34 (25.95)	8	14	12	
		
	N2	50 (38.17)	8	19	23	

Differentiation	Poor + Moderate	124 (94.65)	28	53	43	0.005^#^
		
	well	7 (5.34)	5	2	0	

Perineural Extension	Yes	55 (41.98)	11	22	22	0.538*
		
	No	62 (47.33)	16	27	19	

* Pearson Chi-Square; ^# ^Spearman Correlation (Ordinal by Ordinal)

### Correlation of fascin expression with clinico-pathological parameters of the patients

**Figure 5:**
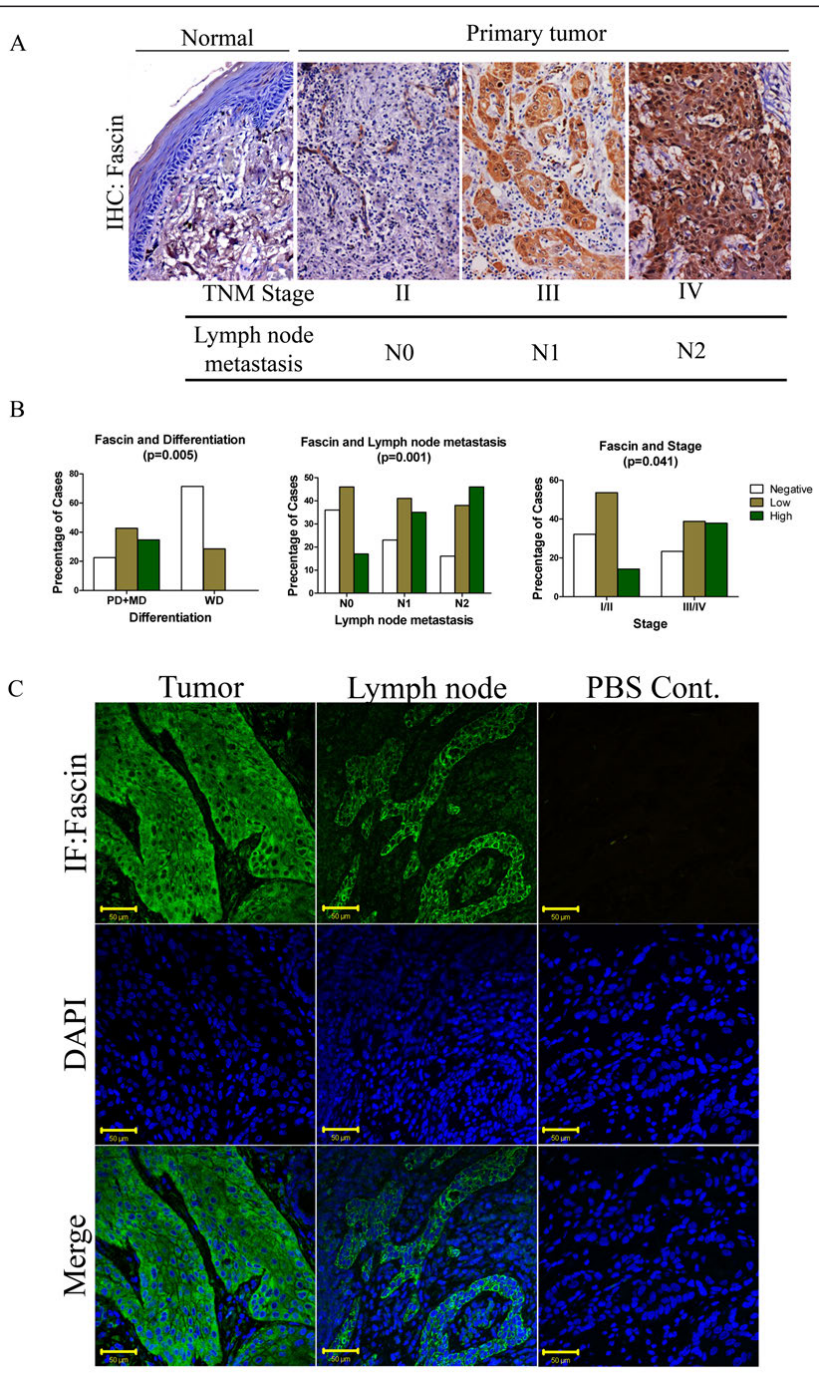
**IHC analysis of fascin levels in human OSCC tissues**. (**A**) Representative images of immunohistochemical staining with antibodies against fascin of paraffin embedded sections of normal different lymph-node metastasis and stage of human OSCC tissues. Sections were counter stained with eosin (Magnification: 200×). (**B**) Histograms showing correlation of fascin expression with clinico-pathological parameters such as differentiation, lymph-node metastasis and tumor stage of OSCC patients. (**C**) Representative images of immunofluoroscence staining with antibodies against fascin of paraffin embedded sections of lymph-node metastasized tumor of human OSCC tissues. Sections were counter stained with DAPI. Scale bars: 50 μm.

To evaluate the significance of fascin expression in OSCC, we investigated the correlation between fascin immunostaining with clinico-pathological parameters of the patients using *Chi *Square test. Higher fascin expression significantly correlated with lymph node metastasis (*P *= 0.001), stage (*P *= 0.041) and differentiation status (*P *= 0.005) (Figure. 5A, B; Table 1). Its expression showed no correlation with other parameters (Additional file 3: Table S2). In order to check whether fascin expression was also seen in lymph node showing tumor metastasis, we have analyzed fascin expression in 6 primary tumors and lymph node obtained from same patient using IHC and IF analysis. Fascin staining was seen in all the primary tumors and corresponding lymph nodes (Figure 5C; Additional file 6: Figure S4).

**Figure 6:**
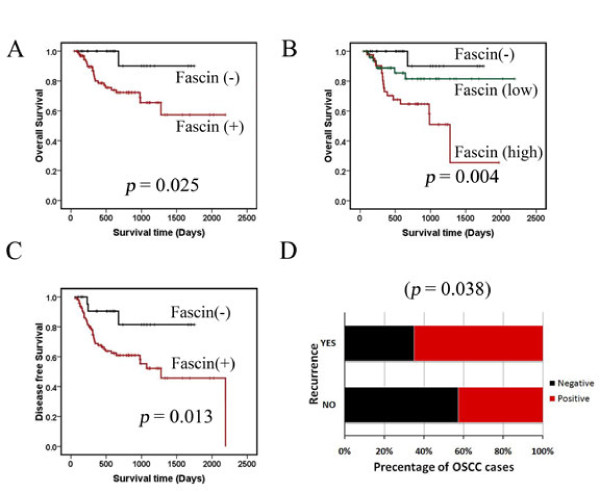
**Correlation of fascin expression with survival rate of OSCC patients using Kaplan-Meier analysis.** (**A** and **B**) Analysis of overall survival in OSCC patients and fascin expression (fascin expression is either negative or positive; *, *p *< 0.025), (fascin expression is negative, low or high; *, *p *< 0.004) (**C**) Analysis of disease free survival times of OSCC patients and fascin (fascin expression is either negative or positive; **, *p *< 0.013) expression. (**D**) Histogram showing percentage of recurrence in fascin positive samples of OSCC patients.

Kaplan-Meier survival analysis was conducted to establish correlation between fascin expression and patient's survival. Overall fascin expression showed negative correlation with patient's survival. Thus higher fascin expression showed inverse correlation with patient's survival (Figure 6A; *P *= 0.025), while low or no fascin expression indicated better survival rate (Figure 6B; *P *= 0.004). In addition, fascin expression significantly correlated with increased recurrence rate (Figure 6D; *P *= 0.038) and shorter disease free survival (Figure 6C; *P *= 0.013).

In our previous study we have found downregulation of fascin and β4-integrin expression in response to K8 knockdown in OSCC cells [28]. Therefore, we have also analyzed K8 (*n *= 131) and β4-integrin (*n *= 123) expression in these samples using IHC (Figure S2) and tried to find out association of these molecules with clinico-pathological parameters of the patients in combination (Additional file 4: Figure S2; Additional file 7: Table S3). Fascin and β4-integrin expression together correlated with lymph node metastasis (*P *= 0.001), stage (*P *= 0.032) and perineural extension (*P *= 0.028) (Additional file 7: Table S3) while fascin and K8 expression together correlated with tumor stage (*P *= 0.020), differentiation (*P *= 0.001), lymph node metastasis (*P *= 0.002), recurrence (*P *= 0.004) (Additional file 7: Table S3). Fascin together with K8 or β4-integrin did not show any correlation with overall/disease free survival of the patients (data not shown).

In summary, our in vitro data demonstrated that fascin overexpression in OSCC derived cells led to increase in cell membrane protrusions, disorganization of cell-cell contacts and alterations in actin cytoskeleton. These cells also showed increased cell-ECM adhesion, cell migration, invasion, and tumorigenicity. In addition, fascin-overexpressed cells demonstrated activation of Akt and MAP kinase. Furthermore, fascin expression significantly correlated with lymph node metastasis, stage and differentiation status in OSCC samples. Fascin expression also associated with poor patient survival.

## Discussion

Understanding the molecular basis of OSCC progression especially invasion and metastasis and identification of potential molecular markers that are contributing to these processes is required to improve the prognosis and the survival of the patients. Invasive and metastatic potential of tumor cells is often associated with reorganization of actin cytoskeleton. Fascin is one such actin bundling protein, found to be up-regulated in several epithelial malignancies including SCC. Its expression has been correlated with aggressiveness of the tumors [12]. In the present study we have investigated the role of fascin in cell motility, invasion and tumorigenicity using in vitro and xenograft in vivo mouse model. We have further tried to understand the clinical significance of fascin expression in prognostication of OSCC patients using IHC analysis of tumor tissues.

The fascin-overexpressed OSCC cells formed longer and thicker microspikes, develop more filopodia and lamellipodia (Figure 1), and showed significant increase in cell motility (Figure 2A, B). Several prior studies have shown that fascin overexpression induce lamellipodia and filopodia formation and enhances cell migratory ability in different cell types [35-37]. These fascin-overexpressed cells also showed disorganization of cell-cell contacts (Figure 3C, D), decrease in E-cadherin and β-catenin levels (Figure 3F). Previously it has been shown that fascin overexpression led to disorganization of cell-cell contacts and reduction in E-cadherin and β-catenin levels in epithelial cells [37]. The observed reduction in total β-catenin levels may be due to loss of cell-cell contacts which intern may lead to its degradation at cell surface (Additional file 2: Figure S1C). Previous reports have also shown reduction or loss of total and/or cell surface levels of β-catenin in OSCC samples and correlated it with aggressiveness of the tumor and poor prognosis [38-40]. Importantly, the anchorage-independent growth and xenograft tumor experiments in NOD-SCID mice indicated that fascin also contributes to the development of primary tumors in OSCC (Figure 5A, B). To our knowledge this is the first report showing the role of fascin in primary tumor development in OSCC. These results are consistent with the previous reports on colon cancer and oesophageal SCC cells [41,42]. Our results further demonstrated significant increase in invasive potential (Figure 2C) in fascin overexpressed cells. Previously, fascin has been shown to promote invasiveness of the colon, breast and esophageal carcinoma derived cells [22,43,44]. Chen et al. have identified migrastatin analogues which inhibit tumor cell migration, invasion and metastasis by blocking fascin activity using in vitro as well as in vivo model system in breast cancer [45]. Furthermore, fascin-overexpressed cells also demonstrated increase in MMP-2 activity (Figure 2D). MMP-2 and MMP-9 are proteolytic enzymes that digest the components of the basement membrane facilitating metastasis of malignant tumors [46]. Our results are consistent with Xie et al. findings and suggest that, increase in MMP-2 activity in response to fascin upregulation would also contribute to increased invasive ability of the carcinoma cells [42]. Thus, our results indicate that fascin overexpression leads to increase in cell motility, tumorigenicity and invasive potential of OSCC cells.

Increased cell migration, invasion, tumorigenicity and loss of E-cadherin expression are often observed during Epithelial Mesenchymal Transition (EMT) [47]. Here fascin overexpressed cells did not show any increase in EMT markers such as snail, slug and vimentin (Figure 3G; Additional file 2: Figure S1B). Our results are in agreement with the previous report, in which Vignjevic et al. have shown that fascin mediated tumor cell migration and invasion did not involve EMT in human colon cancer [48]. These results together suggest that fascin may promote cell migration and invasion of tumor cells through collective cell migration by filopodia formation [49] in OSCC.

Interactions between cells and their extracellular matrix play an important role in tissue organisation and also influence many aspects of cell behaviour including cell shape and cell migration [50-52]. During cell migration, Thrombpspondin-1 induces cross-linking of fascin and F-actin that leads to formation of F-actin based cell protrusions. Fibronectin triggers phosphorylation of fascin at S39, leading to rapid loss of fascin from actin-based structures during initial cell spreading, in a protein kinase Ca (PKCa)-dependent process [53,54]. Here, fascin overexpression facilitated cell adhesion to fibronectin, laminin and other ECM molecules in OSCC cells. Previously, it has been shown that fascin may be involved in cell-ECM interaction through integrin [55]. Previous reports also suggest that fascin regulates focal adhesion disassembly and activation of FAK [56]. We did not observe any consistent change in α6-integrin, β4-integrin and phosphorylated FAK levels in all the fascin overexpressed clones (Figure 3B). This suggests that α6β4-integrin-FAK-mediated signalling may not be involved in fascin dependent cell-ECM interaction in OSCC.

Overexpression of fascin is often associated with increased cell proliferation in different types of carcinomas [42,43,57]. Our results have shown increase in cell proliferation in response to fascin overexpression which was accompanied with increased cell proliferation marker PCNA (Figure 4D). In addition, upregulation of PI3K and MAP kinase pathways was also observed in fascin-overexpressed cells (Figure 4D, E). PI3-K and MAP kinase pathways are independently known to regulate cell proliferation and migration in epithelial cells and upregulation of these signalling pathways has been documented in carcinomas including OSCC [58-60]. Further, the role of MAPKs in the regulation of MMP expression in carcinoma cells has also been well-studied [61]. It has been shown that inhibition of p-ERK1/2 may lead to a reduction in the expression of MMP-2 and invasive ability of tumor cells [62,63]. These results suggest that fascin possibly regulates MMPs activity and invasiveness through MAPKs pathway. Previously it has been reported that Akt activation is a significant prognostic indicator for OSCC [64]. Taken together our results suggest that apart from regulating actin polymerization and thereby cell motility, fascin may also activate PI3K and MAP kinase signalling pathways in tumor cells. These events subsequently promote tumor progression.

Next we investigated the clinical significance of fascin expression in prognostication of OSCC patients. Correlation of fascin expression with lymph node metastasis and its presence (Figure 5; Table 1) in lymph node tissues showing tumor metastasis (Figure 5C) suggest that fascin may facilitate movement of tumor cells from the primary site to the lymph node. Lymph node metastasis is widely accepted as one of the major prognostic factors in OSCC patients and it's presence is associated with a decrease in overall survival and higher recurrence rates [6,65-68]. High fascin expression was seen in 17% cases where lymph node metastasis was not detected (N0). It will be interesting to follow these cases further to understand whether fascin expression is indicative of "submicroscopic" occult metastasis which is known to have impact on patient survival [65,67,69]. Furthermore, fascin expression was not detected in any of the well differentiated OSCC (Figure 5B; Table 1) and higher fascin expression was mostly found in higher stage tumors (Figure 5A, B; Table 1). A large number of studies have demonstrated that disease staging has a crucial influence on the outcome [66,70]. A number of studies have also shown significant correlation between lower histologic differentiation and poorer prognosis [6,66,70]. We were further able to show correlation between fascin expression and poor patient survival (Figure 6A, B). On the other hand fascin non-expression was indicative of disease free survival and decreased chance of recurrence (Figure 6C, D). Although, Chen et al. have demonstrated correlation of fascin levels with tumor size, lymph node metastasis and staging, they have not reported correlation study with survival of the patients [27]. Lee et al. have also shown correlation of fascin expression with nodal metastasis, tumor recurrence and poor patient's survival in 49 OSCC samples [27,71]. In this study, we did not observe correlation of fascin expression with tumor size but we could show correlation with differentiation status. Thus apart from confirming the findings of previous studies on larger sample size, we could show its relation with disease free survival and confirm its expression in lymph node metastasis. Thus our results demonstrate prognostic value of fascin expression in OSCC.

Recently, we have shown that knockdown of K8 in an OSCC derived cell line resulted in reduced fascin and β4-integrin levels which correlated with decreased tumorigenicity, migration and invasiveness of these cells [28]. Therefore, we further analyzed association of fascin in combination with K8 and β4-integrin expression with clinico-pathological parameters of the patients. Fascin and K8 expression together correlated with tumor stage, differentiation status, lymph node metastasis and recurrence while fascin and β4-integrin expression together correlated with differentiation status, lymph node metastasis and per neural tumor extension (Additional file 7: Table S3). We did not observe correlation between expression of all these proteins (fascin, β4-integrin and K8) together with patient survival (data not shown) possibly because of the smaller sample size. A study with larger sample size is required to highlight the significance of their expression together in prognostication of OSCC.

## Conclusions

In conclusion, fascin promotes tumor cell progression and modulates tumor associated signalling pathways such as Akt and MAP kinase in OSCC. The precise molecular mechanism of fascin dependent regulation of Akt and MAPK in OSCC needs further investigation. In addition, increased fascin levels in human OSCC significantly correlated with stage, lymph node metastasis and poor patient survival. Thus, the current study not only has identified a novel mechanism of fascin dependent tumor progression but also illustrated a prognostic value of fascin in OSCC. Furthermore, actin cytoskeleton associated protein such as fascin can be explored as new therapeutic target for human oral cancer. Potential drugs such as migrastatin analogues targeting fascin may prove to be effective in the prevention or treatment of higher grade and metastatic oral tumors.

## Authors' contributions

HA conceived and carried out experiment, analyzed the data and drafted the manuscript. AVB, PG, SS, PPD and LS carried out experiments. SSS and PG analyzed the IHC staining. SK performed statistical analysis. DAC, AKD and SK performed clinico-pathological examination of the tumor samples. RG and SND supported the experiments. MMV designed the study and prepared it for publication. All authors were involved in manuscript preparation and had final approval of the submitted version.

## Pre-publication history

The pre-publication history for this paper can be accessed here:

http://www.biomedcentral.com/1471-2407/12/32/prepub

## Supplementary Material

Additional file 1 **Table S1. **Clinico-pathological parameters of the OSCC patients. Click here for file

Additional file 2 **Figure S1. **(**A**) Cell morphology (shape) of fascin-overexpressed and vector control clone analyzed by phase contrast microscopy. Scale bar: 100 μm. (**B**) Western blot analysis of fascin-overexpressed (AW-Fascin-1 and AW-Fascin-2) and vector control clones (AW-GFP-Cont) with antibody to vimentin. β-actin was used as a loading control. (**C**) Representative confocal images of b-catenin and E-cadherin staining in stable AW-Fascin-1, AW-Fascin-2 and AW-GFP-Cont clones. Scale bars: 10 μm. (**D**) Representative image of the size and number of colonies formed in soft agar of the indicated clones. Scale bar: 100 μm. Click here for file

Additional file 3 **Table S2. **The Fascin overexpressed clones show a decrease in cell-cell adhesion. Cell adhesion was measured by the hanging drop assay as described. 2 × 10^4 ^cells of the indicated clones were resuspended in 35 μl of complete medium on the lid of a 24 well dish. 16 h later the cells were fixed and the number and area of aggregates in fifteen fields was measured. The numbers of aggregates of different sizes are shown. Click here for file

Additional file 4 **Figure S2. **Representative images of haematoxylin and eosin along with immunohistochemical staining with antibodies against fascin, K8 and β4-integrin on paraffin embedded sections of human in oral tumors (**A**) and non malignant tissues (**B**). Sections were counter stained with eosin (Magnification: 200×). Click here for file

Additional file 5 **Figure 3. **(**A**) Representative images of immunofluorescence staining with antibodies against β4-integrin and K1 of paraffin embedded sections of non malignant oral tissues. Sections were counter stained with DAPI. Scale bar: 50 μm. (**B**) Representative images of immunofluoroscence staining with antibodies against fascin, β4-integrin and K14 of paraffin embedded sections of human oral tumors and fascin IF stainging in non malignant oral tissues. Sections were counter stained with DAPI. Scale bar: 50 μm. Click here for file

Additional file 6 **Figure S4. **Representative images of IHC staining with antibodies against fascin on paraffin embedded sections o f primary tumor and lymph node metastasized tumor of human OSCC tissues. Sections were counter stained with eosin (Magnification: 200×). Click here for file

Additional file 7 **Table S3. **Correlations of fascin in combination with K8 and β4-integrin expression with clinico-pathological parameters of the OSCC patients. Click here for file

Additional file 8 **Figure R1. **(**A** and **B**)Western blot analysis of fascin-overexpressed (AW-Fascin-1 and AW-Fascin-2) and vector control clones (AW-GFP-Cont) with antibodies to α6-integrin, β4-integrin and pFAK. β-actin and FAK were used as loading control respectively. Densitometric analysis for the quantification of level of α6-integrin, β4-integrin and pFAK obtained from western blot of the indicated clones. (**C **and **D**) Western blot analysis of fascin-overexpressed (AW-Fascin-1 and AW-Fascin-2) and vector control clones (AW-GFP-Cont) with antibodies to fascin. β-actin was used as a loading control. Quantification of levels of fascin in fascin-overexpressed (AW-Fascin-1 and AW-Fascin-2) and vector control (AW-GFP-Cont) clones using densitometric analysis. Click here for file
