# Supplementary material for: Fascin overexpression promotes neoplastic progression in oral squamous cell carcinoma
Source: BMC Cancer. 2012 Jan 20;12:32. doi: 10.1186/1471-2407-12-32 (PMC3329405; doi:10.1186/1471-2407-12-32)
Supplement: Additional file 1 — Table S1. Clinico-pathological parameters of the OSCC patients. [file 1471-2407-12-32-S1.DOC]

**Table S1: Clinico-pathological parameters of the OSCC patients.**

| **Clinico-pathological parameters** | | **No of cases (131)** | **Percentage** |
| --- | --- | --- | --- |
| **Age (Years)** | **<50** | 75 | 57.25 |
|  | **≥50** | 56 | 42.75 |
| **Sex** | **Male** | 102 | 77.87 |
|  | **Female** | 29 | 22.14 |
| **Location** | **Tongue** | 61 | 46.56 |
|  | **BM** | 70 | 53.44 |
| **Thickness** | **<2cm** | 92 | 70.23 |
|  | **≥2cm** | 39 | 29.77 |
| **Stages** | **I** | 11 | 8.40 |
|  | **II** | 17 | 12.98 |
|  | **III** | 18 | 13.74 |
|  | **IV** | 85 | 64.89 |
| **Tumor**  **Size** | **<T3** | 48 | 36.65 |
|  | **≥T3** | 83 | 63.35 |
| **Node Status** | **NO** | 47 | 35.88 |
|  | **N1** | 34 | 25.95 |
|  | **N2** | 50 | 38.17 |
| **Differentiation** | **Poor+ Moderate** | 124 | 94.65 |
|  | **well** | 7 | 5.34 |
| **Bone** | **Positive** | 33 | 25.19 |
|  | **Negative** | 69 | 52.67 |
| **Perineural Invasion** | **Yes** | 28 | 21.37 |
|  | **No** | 92 | 70.23 |
| **Lympho vascular invasion** | **Yes** | 3 | 2.29 |
|  | **No** | 112 | 85.50 |
| **Perineural Extension** | **Yes** | 55 | 41.98 |
|  | **No** | 62 | 47.33 |
| **Cut Margin** | **Free** | 118 | 90.08 |
|  | **Closed** | 8 | 6.11 |
|  | **Invasion** | 5 | 3.82 |
| **Recurrence** | **Yes** | 49 | 38.58 |
|  | **No** | 78 | 61.42 |
| **Skin** | **Yes** | 11 | 8.40 |
|  | **No** | 68 | 51.91 |
