# Supplementary material for: Fascin overexpression promotes neoplastic progression in oral squamous cell carcinoma
Source: BMC Cancer. 2012 Jan 20;12:32. doi: 10.1186/1471-2407-12-32 (PMC3329405; doi:10.1186/1471-2407-12-32)
Supplement: Additional file 2 — Figure S1. (A) Cell morphology (shape) of fascin-overexpressed and vector control clone analyzed by phase contrast microscopy. Scale bar: 100 μm. (B) Western blot analysis of fascin-overexpressed (AW-Fascin-1 and AW-Fascin-2) and vector control clones (AW-GFP-Cont) with antibody to vimentin. β-actin was used as a loading control. (C) Representative confocal images of b-catenin and E-cadherin staining in stable AW-Fascin-1, AW-Fascin-2 and AW-GFP-Cont clones. Scale bars: 10 μm. (D) Representative image of the size and number of colonies formed in soft agar of the indicated clones. Scale bar: 100 μm. [file 1471-2407-12-32-S2.PDF]

Fig S1

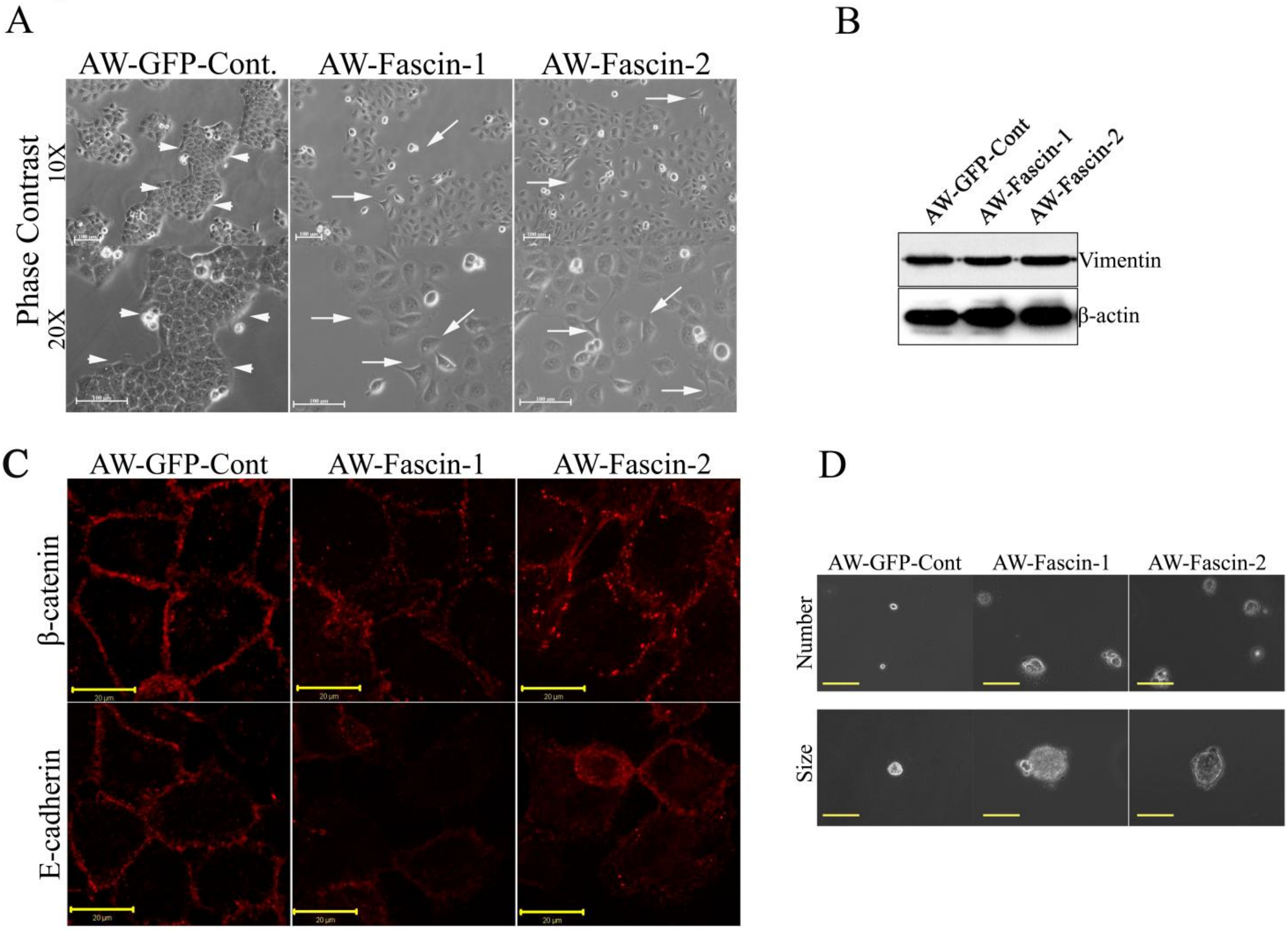

Figure S1: (A) Cell morphology (shape) of fascin-overexpressed and vector control clone analysed by phase contrast microscopy. Scale bar: 100µm. **(B)** Western blot analysis of fascin-overexpressed (AW-Fascin-1 and AW-Fascin-2) and vector control clones (AW-GFP-Cont) with antibody to vimentin. β-actin was used as a loading control. **(C)** Representative confocal images of β-catenin and E-cadherin staining in stable AW-Fascin-1, AW-Fascin-2 and AW-GFP-Cont clone. Scale bars: 10µm. **(D)** Representative image of the size and number of colonies formed in soft agar of the indicated clones. Scale bar: 100µm.
