# Supplementary material for: Fascin overexpression promotes neoplastic progression in oral squamous cell carcinoma
Source: BMC Cancer. 2012 Jan 20;12:32. doi: 10.1186/1471-2407-12-32 (PMC3329405; doi:10.1186/1471-2407-12-32)
Supplement: Additional file 3 — Table S2. The Fascin overexpressed clones show a decrease in cell-cell adhesion. Cell adhesion was measured by the hanging drop assay as described. 2 × 10^4^ cells of the indicated clones were resuspended in 35 μl of complete medium on the lid of a 24 well dish. 16 h later the cells were fixed and the number and area of aggregates in fifteen fields was measured. The numbers of aggregates of different sizes are shown. [file 1471-2407-12-32-S3.DOC]

**Table S2. The Fascin overexpressed clones show a decrease in cell-cell adhesion.** Cell adhesion was measured by the hanging drop assay as described. 2 x 10^4^ cells of the indicated knockdown clones were resuspended in 35μl of complete medium on the lid of a 24 well dish. 16 hours later the cells were fixed and the number and area of aggregates in fifteen fields was measured. The numbers of aggregates of different sizes are shown.

| Construct | Aggregates per field | | | |
| --- | --- | --- | --- | --- |
|  | >10^5^μm^2^ | 10^5^-2x10^4^ μm^2^ | <2x10^4^-1x10^4^ μm^2^ | <10^4^-3.5x10^3^ μm^2^ |
| AW-GFP-Cont | 3 | 12 | 10 | 0 |
| AW-Fascin-1 | 0 | 0 | 0 | 25 |
| AW-Fascin-2 | 0 | 0 | 0 | 25 |
