# Supplementary material for: Fascin overexpression promotes neoplastic progression in oral squamous cell carcinoma
Source: BMC Cancer. 2012 Jan 20;12:32. doi: 10.1186/1471-2407-12-32 (PMC3329405; doi:10.1186/1471-2407-12-32)
Supplement: Additional file 4 — Figure S2. Representative images of haematoxylin and eosin along with immunohistochemical staining with antibodies against fascin, K8 and β4-integrin on paraffin embedded sections of human in oral tumors (A) and non malignant tissues (B). Sections were counter stained with eosin (Magnification: 200×). [file 1471-2407-12-32-S4.PDF]

Fig S2

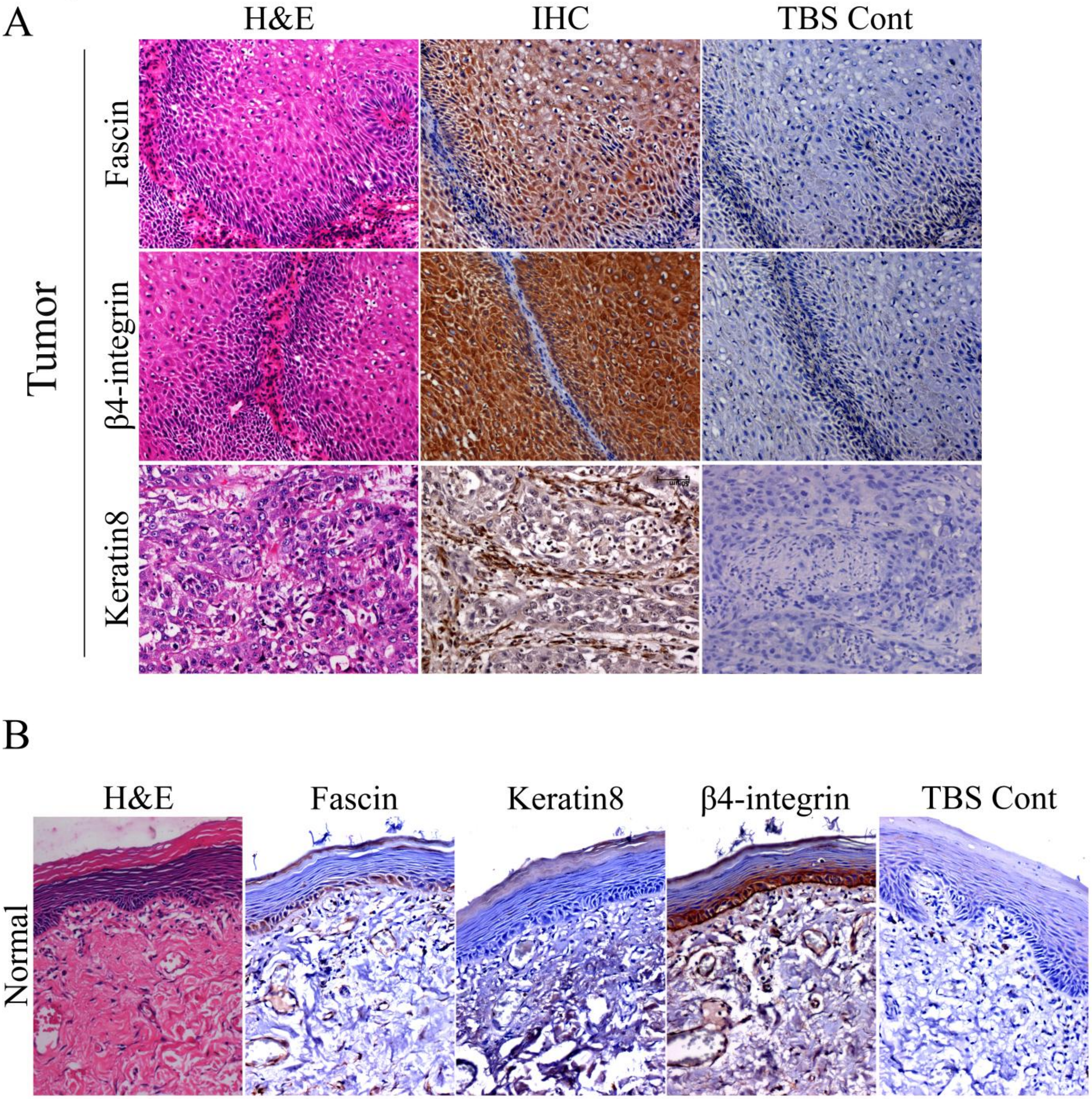

Figure S2: Representative images of haematoxylin and eosin along with immunohistochemical staining with antibodies against fascin, K8 and β4-integrin on paraffin embedded sections of human in oral tumor **(A)** and non malignant tissues **(B)**.
