# Supplementary material for: Fascin overexpression promotes neoplastic progression in oral squamous cell carcinoma
Source: BMC Cancer. 2012 Jan 20;12:32. doi: 10.1186/1471-2407-12-32 (PMC3329405; doi:10.1186/1471-2407-12-32)
Supplement: Additional file 5 — Figure 3. (A) Representative images of immunofluorescence staining with antibodies against β4-integrin and K1 of paraffin embedded sections of non malignant oral tissues. Sections were counter stained with DAPI. Scale bar: 50 μm. (B) Representative images of immunofluoroscence staining with antibodies against fascin, β4-integrin and K14 of paraffin embedded sections of human oral tumors and fascin IF stainging in non malignant oral tissues. Sections were counter stained with DAPI. Scale bar: 50 μm. [file 1471-2407-12-32-S5.PDF]

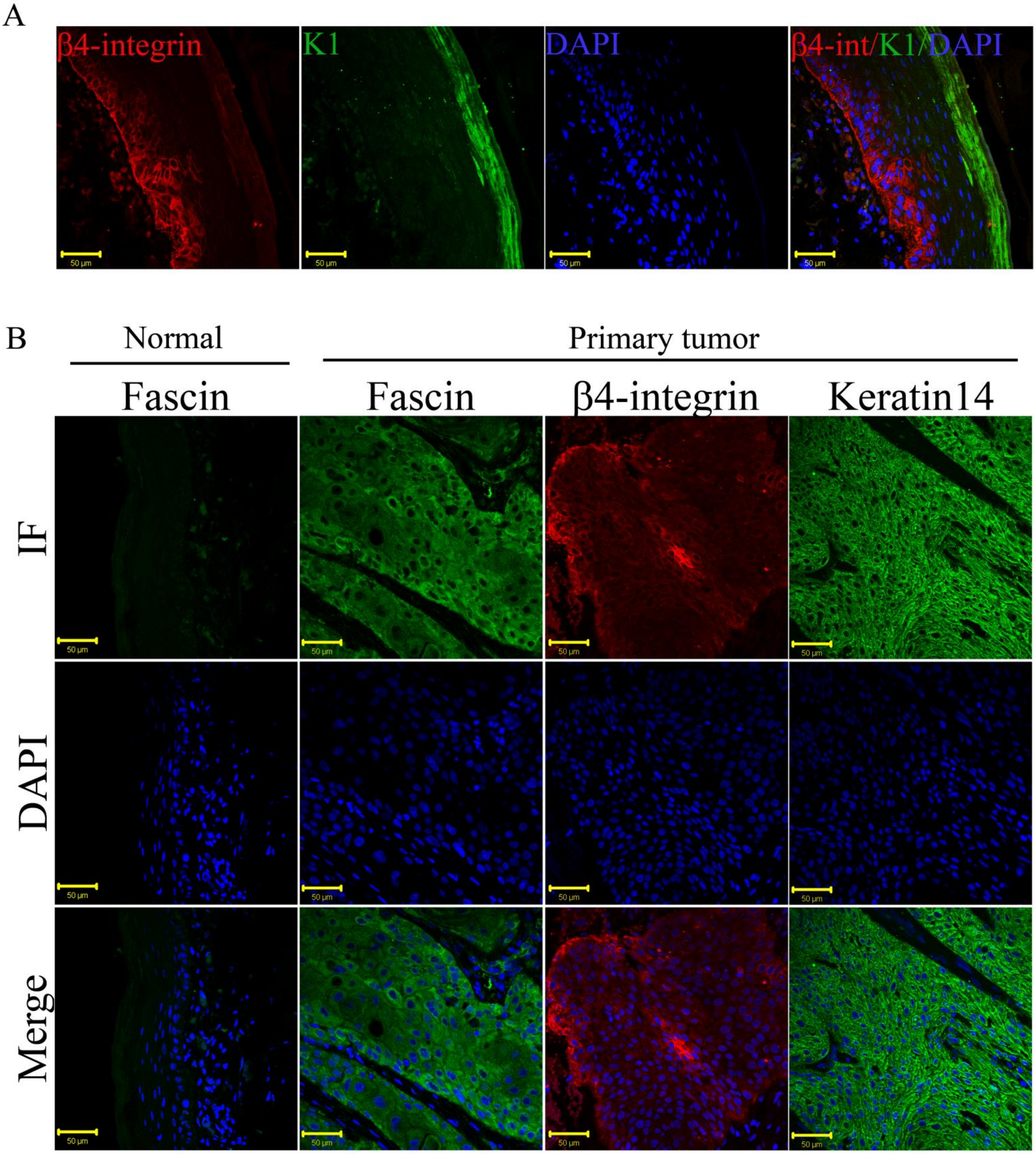

Figure S3: (A) Representative images of immunofluoroscence staining with antibodies against fascin, β4-integrin and K14 of paraffin embedded sections of human oral tumor and fascin IF staining in non malignant tissues **(B)**. Representative images of immunofluoroscence staining with antibodies against β4-integrin and K1 of paraffin embedded sections of non malignant oral tissues.
