# Supplementary material for: Fascin overexpression promotes neoplastic progression in oral squamous cell carcinoma
Source: BMC Cancer. 2012 Jan 20;12:32. doi: 10.1186/1471-2407-12-32 (PMC3329405; doi:10.1186/1471-2407-12-32)
Supplement: Additional file 6 — Figure S4. Representative images of IHC staining with antibodies against fascin on paraffin embedded sections o f primary tumor and lymph node metastasized tumor of human OSCC tissues. Sections were counter stained with eosin (Magnification: 200×). [file 1471-2407-12-32-S6.PDF]

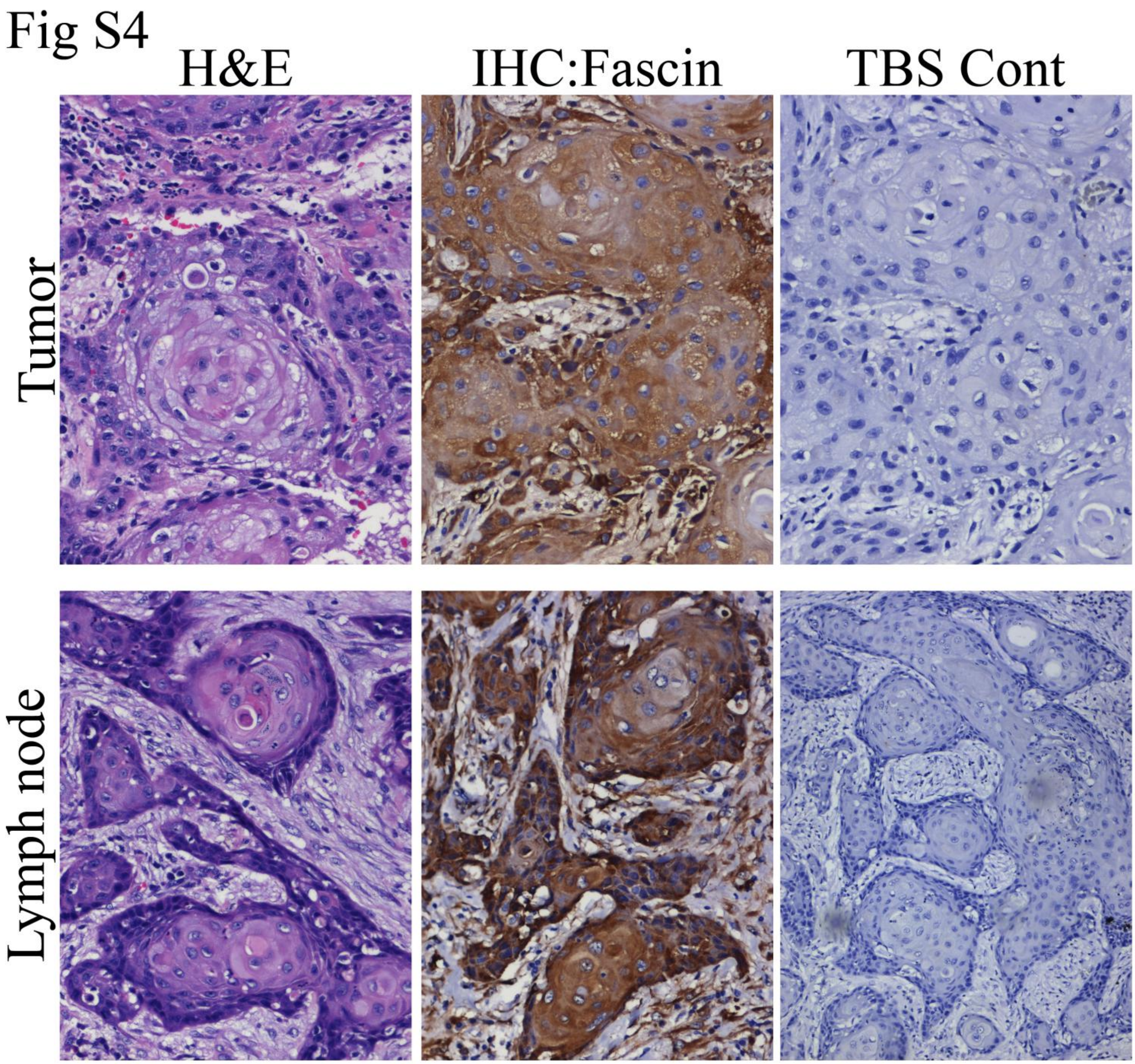

Figure S4: Representative images of IHC staining with antibodies against fascin on paraffin embedded sections of primary tumor and lymph node metastasized tumor of human OSCC tissues.
