# Supplementary material for: Fascin overexpression promotes neoplastic progression in oral squamous cell carcinoma
Source: BMC Cancer. 2012 Jan 20;12:32. doi: 10.1186/1471-2407-12-32 (PMC3329405; doi:10.1186/1471-2407-12-32)
Supplement: Additional file 7 — Table S3. Correlations of fascin in combination with K8 and β4-integrin expression with clinico-pathological parameters of the OSCC patients. [file 1471-2407-12-32-S7.DOC]

**Table S3: Correlations of fascin in combination with K8 and β4-integrin expression with clinico-pathological parameters of the OSCC patients.**

* Pearson Chi-Square; ^#^ Spearman Correlation (Ordinal by Ordinal)

| **Clinico-pathological parameter** | | **FASCIN AND β 4 INTEGRIN** | | | | | **FASCIN AND K8** | | | | |
| --- | --- | --- | --- | --- | --- | --- | --- | --- | --- | --- | --- |
|  |  | **n**  **(123)** | **EXPRESSION** | | | ***P*-value** | **n**  **(131)** | **EXPRESSION** | | | ***P*-value** |
|  |  |  | **fascin(+)**  **β4(+)** | **fascin(+)**  **β4 (-)** | **fascin(-)**  **β4 (+) or**  **fascin(-)**  **β4 (-)** |  |  | **Fascin (+)**  **K8 (+)** | **fascin(+)**  **K8 (-)** | **fascin(-)**  **K8 (+) or fascin(-)**  **K8 (-)** |  |
| **Age (Years)** | **<50** | 71 | 44 | 13 | 14 | 0.202* | 75 | 48 | 16 | 15 | 0.285* |
|  | **≥50** | 52 | 24 | 12 | 16 |  | 56 | 22 | 10 | 18 |  |
| **Sex** | **Male** | 99 | 57 | 19 | 23 | 0.582* | 102 | 58 | 19 | 25 | 0.693* |
|  | **Female** | 24 | 11 | 6 | 7 |  | 29 | 14 | 7 | 8 |  |
|  | **≥2cm** | 37 | 22 | 7 | 8 |  | 39 | 23 | 8 | 8 |  |
| **Stages** | **I/II** | 27 | 9 | 10 | 8 | **0.032^#^** | 28 | 9 | 10 | 9 | **0.020^#^** |
|  | **III/IV** | 96 | 59 | 15 | 22 |  | 103 | 63 | 16 | 24 |  |
| **Tumor**  **Size** | **T1/T2** | 46 | 21 | 14 | 11 | 0.246^#^ | 48 | 21 | 14 | 13 | 0.124^#^ |
|  | **T3/T4** | 77 | 47 | 11 | 19 |  | 83 | 51 | 12 | 20 |  |
| **Node Status** | **NO** | 43 | 13 | 15 | 15 | **0.001**^#^ | 47 | 17 | 13 | 17 | **0.002**^#^ |
|  | **N1** | 33 | 20 | 5 | 8 |  | 34 | 21 | 5 | 8 |  |
|  | **N2** | 47 | 35 | 5 | 7 |  | 50 | 34 | 8 | 8 |  |
| **Differentiation** | **Poor+ Moderate** | 116 | 66 | 24 | 26 | 0.068^#^ | 124 | 72 | 24 | 28 | **0.001**^#^ |
|  | **well** | 7 | 2 | 1 | 4 |  | 7 | 0 | 2 | 5 |  |
|  | **No** | 107 | 59 | 24 | 24 |  | 112 | 61 | 24 | 27 |  |
| **Perineural Extension** | **Yes** | 52 | 36 | 6 | 10 | **0.028*** | 55 | 35 | 9 | 11 | 0.323* |
|  | **No** | 59 | 27 | 17 | 15 |  | 62 | 31 | 15 | 16 |  |
| **Recurrence** | **Yes** | 48 | 29 | 12 | 7 | 0.213* | 49 | 25 | 17 | 7 | **0.004*** |
|  | **No** | 72 | 39 | 13 | 20 |  | 78 | 46 | 9 | 23 |  |
