# Supplementary material for: Fascin overexpression promotes neoplastic progression in oral squamous cell carcinoma
Source: BMC Cancer. 2012 Jan 20;12:32. doi: 10.1186/1471-2407-12-32 (PMC3329405; doi:10.1186/1471-2407-12-32)
Supplement: Additional file 8 — Figure R1. (A and B)Western blot analysis of fascin-overexpressed (AW-Fascin-1 and AW-Fascin-2) and vector control clones (AW-GFP-Cont) with antibodies to α6-integrin, β4-integrin and pFAK. β-actin and FAK were used as loading control respectively. Densitometric analysis for the quantification of level of α6-integrin, β4-integrin and pFAK obtained from western blot of the indicated clones. (C and D) Western blot analysis of fascin-overexpressed (AW-Fascin-1 and AW-Fascin-2) and vector control clones (AW-GFP-Cont) with antibodies to fascin. β-actin was used as a loading control. Quantification of levels of fascin in fascin-overexpressed (AW-Fascin-1 and AW-Fascin-2) and vector control (AW-GFP-Cont) clones using densitometric analysis. [file 1471-2407-12-32-S8.PDF]

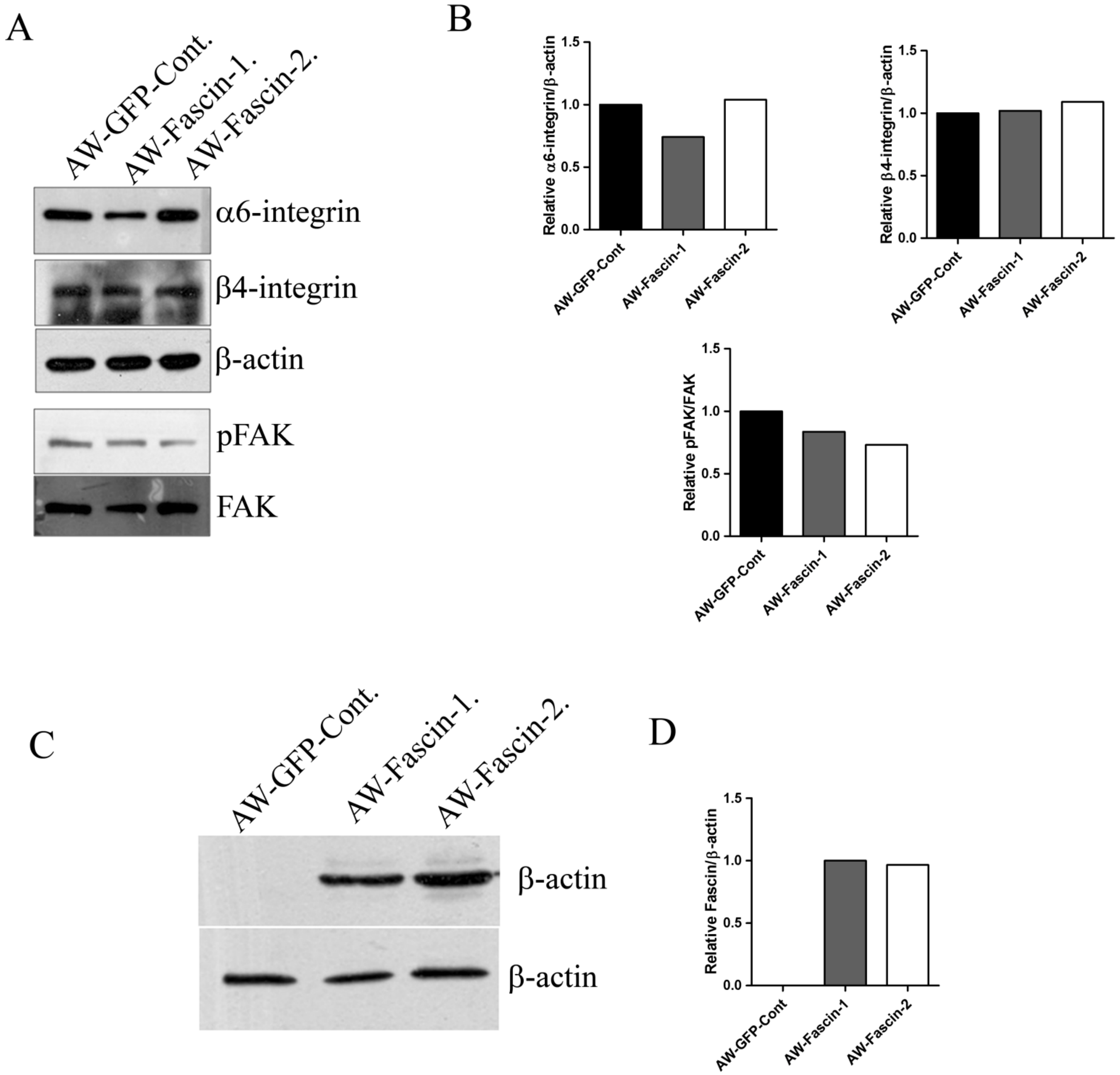

Figure R1: (A and B) Western blot analysis of fascin-overexpressed (AW-Fascin-1 and AW-Fascin-2) and vector control clones (AW-GFP-Cont) with antibodies to α6-integrin, β4-integrin and pFAK. β-actin and FAK were used as a loading control. Quantification of levels of α6-integrin, β4-integrin and pFAK obtained from western blot in the clones using densitometry. **(C and D)** Western blot analysis of fascin-overexpressed (AW-Fascin-1 and AW-Fascin-2) and vector control clones (AW-GFP-Cont) with antibodies to fascin. β-actin was used as a loading control. Quantification of levels of fascin in fascin-overexpressed (AW-Fascin-1 and AW-Fascin-2) and vector control clones (AW-GFP-Cont) using densitometry.
